# Effects of Complex Fertilizers on the Properties of Grey Forest Heavy Loamy Soil

**DOI:** 10.1155/2024/2763147

**Published:** 2024-03-07

**Authors:** Sergey Gaidar, Anastasia Kazak, Alina Barchukova, Andrey Kozlov

**Affiliations:** ^1^Department of Materials Science and Engineering Technology, Russian State Agrarian University-Moscow Timiryazev Agricultural Academy, Moscow, Russia; ^2^Department of Biotechnology and Breeding in Crop Production, Federal State Budgetary Educational Institution of Higher Education Northern Trans-Ural State Agricultural University, Tyumen, Russia; ^3^Department of Microbiology and Immunology, Russian State Agrarian University-Moscow Timiryazev Agricultural Academy, Moscow, Russia

## Abstract

The study's main aim was to evaluate the effects of complex mineral fertilizers on the complex properties of heavy loam soils in the grey forests of Russia in terms of applying individual soil nutrition components from experiments with fodder beets. This study employed a rigorous and systematic approach to accomplish the defined goal. Specifically, the research was conducted within a seven-field crop rotation system, with fodder beets serving as the primary experimental crop. In addition, a model experiment resembling a vegetation trial was undertaken, incorporating seven distinct schemes involving various types of fertilizers. This design facilitated the evaluation of the effectiveness of each fertilizer type. The study results demonstrate that complex fertilizers impact the soil's chemical and biophysical parameters. Soil acidity decreases through the use of complex, high-nitrogen fertilizers. Major chemical nutrients (nitrogen, phosphorus, and potassium) in plant biomass and soil also have a high degree of transition. It is explained by the effects of combining elements on the destruction intensity of the crystalline lattice in the basic structures of potassium, phosphorus, and nitrogen. There is also evidence that complex fertilizers can improve humus quality and replenish its reserves. All the aforementioned impacts of complex fertilizers on the crop contribute to the high productivity and yield of forage beet. The results of the study may help optimize the fertilization process, improve the quality and quantity of agricultural products, as well as increase soil fertility, and reduce the negative impact of agrochemicals on the environment.

## 1. Introduction

Despite enormous technological advances, the modern population and ancestors satisfied their food requirements using the soil as the source of plant nutrients. A growing population calls for more intensive agricultural land use. With each new crop, the equilibrium of the soil's chemical elements and properties changes, mineral reserves exhaust, and fertility decreases.

The sustainable production of crops depends on the continuous renewal of the macroelements and microelements in the soil. To avoid losing the most valuable food source, people need to replenish the necessary nutrients through artificial fertilizer application in the soil. From the variety of existing agricultural fertilizers, the most efficient for almost every type of soil and crop have complex fertilizers [1, 2].

Complex fertilizers combine several chemically active elements (nitrogen, potassium, phosphorus, and other micro- and macroelements such as magnesium, calcium, and sulfur). Plants are therefore provided with balanced nutrition for growth, development, and high yield [3]. However, insufficient or, on the contrary, excessive fertilizer application does not guarantee constant yield growth, which can lead to low efficiency in nutrient use. Considering soils' availability and nutrient content provides the basis for theoretically justifying recommendations to agricultural enterprises for applying fertilizers. Uncontrolled fertilization can have devastating consequences on soil conditions. In particular, these include contamination and poisoning of crops, water bodies, and animals, increased soil acidity or alkalinity, and soil degradation, and even significant changes in the local climate. Modern farm businesses seek the most efficient use of fertilizers, which reduces their production costs and adverse environmental effects [4, 5].

The impact of complex fertilizers on soil properties requires special attention. Moreover, the increase in optimum fertilizer doses for the main crops in the region is a topical problem for central agricultural countries, such as Russia, Ukraine, Kazakhstan, and China. The study of processes that occur in soils after the artificial introduction of complex fertilizers can play an essential role in raising domestic agriculture to a new level in terms of quantity and quality of agricultural crop yield. Even though applying fertilizers with multielement complexes attracts more and more attention from agricultural producers and consumers, its impact on the composition of soils and plants remains insufficiently studied and formed for many regions of Russia. Thus, there is relatively little experimental evidence of the impact and degree of complex fertilizer influence on the basic soils of agroecosystems in Russia.

The study of complex fertilizer effects on the properties of heavy loam soils in grey forests is an urgent and actual research topic. These soils have specific properties and represent an important natural resource. At the same time, the use of fertilizers to increase the yield and quality of crops is an important task of modern agriculture. It is also important to note that the data on the effect of complex fertilizers on the properties of heavy loam soils in grey forests would reveal the effectiveness of using such fertilizers to increase soil fertility and crop yield. In addition, the study can help identify the relationship between the content of complex fertilizers and changes in the physicochemical properties of the soil.

The originality of the study on the effect of complex fertilizers on the properties of heavy loam soils in grey forests is justified by several factors:

- The uniqueness of the research object: heavy loam soils in grey forests have specific properties related to their genesis and distribution. These soils are found in various regions of the world, but their study has not been widely conducted.
- The use of complex fertilizers: the study considers complex fertilizers that contain several types of nutrients. The effect of these fertilizers on soil properties may differ from that of individual fertilizers. This problem has not been sufficiently studied before.

## 2. Literature Review

### 2.1. Characteristics of Complex Fertilizers

Studies by local and foreign scientists based on long-term field experiments show that complex fertilizers are significantly more effective than simple fertilizers. Complex fertilizers allow replenishing balanced doses of trace elements. At the same time, a one-time application of fertilizers in one cultivation operation may considerably reduce transport and storage costs for mechanization and packaging.

Complex fertilizers contain two or more components of soil nutrients (N, P, and K). They are produced in two forms: liquid and solid. Depending on the chemical composition and manufacturing method, there are complex, mixed, and complex-mixed fertilizers.

Complex solid fertilizers are mainly produced from orthophosphoric acid at a concentration of up to 54%. Complex fertilizers such as diammophos, nitrophoska, ammophos, nitrophos, nitroammophoska, crystallines, superphoska, carboammophos, nitrodiammophos, potassium nitrate, carboammophoska, calcium metaphosphate, ammonium metaphosphate, potassium monophosphate, ammonium polyphosphate, and magnesium ammonium phosphate are the most common solid complex fertilizers [6].

The use of solid phosphorus-containing complex fertilizers has a significant disadvantage. When applying them to the soil, anion H_2_PO_4_^2-^ binds to K_3_PO_4_, K_2_HPO_4_, KH_2_PO_4_ (potassium phosphates), and Mg, decreasing the availability of these elements for plants. In particular, this process rapidly occurs on residual carbonate soils and shallow and no-till soils. On a chemical level, soil anions are bound due to the movement of calcium carbonates to upper soil layers. Therefore, it is more effective to use liquid complex fertilizers (LCF) based on H_4_P_2_O_7_ (pyrophosphoric acid) in the heavy loam soils in grey forests of Russia. It has no chemical interaction with soil magnesium and potassium carbonates, as they contain nitrogen and phosphorus in their composition [7, 8].

Scientific studies have shown the clear advantages of liquid complex fertilizers, compared with solid forms, which have lower solubility in soil and water. Liquid complex fertilizers create the most effective nutrient regime for plants, being evenly distributed in the soil's surface layer when applied. In field experiments with spreaders, the uneven distribution of solid fertilizers can reach 73%, frequently leading to a significant shortfall in the yield of crops. However, when applying complex liquid fertilizers with sprayers, there was no intrasoil irregularity of nutrition elements in field experiments [9, 10].

### 2.2. Effect of Complex Fertilizers

According to U.N. data, the use of liquid complex fertilizers (LCF) worldwide is uneven. Thus, the Netherlands consumes about 260 kg per 1 ha, the U.K.—245 kg, Germany—205 kg, Belarus—200 kg, and Ukraine—173 kg. In the last five years, there have been trends to increase the use of liquid complex fertilizers in Russia, namely nitrogen fertilizers, such as KAS and ammonia water. Their use in the fields of Russia is more than 12% [10].

Studies conducted by agrochemists in Ukraine in 2019 showed the effectiveness of using carbamide-ammonia mixtures and LCFs 11–37 in the early spring fertilization of winter wheat. Sorghum was selected as a precursor crop in the rotation based on the minimum tillage of clay loam soils against the background of applying N_40_P_40_K_40_ as the complex fertilizer. Field experiments demonstrated the effectiveness of adding LCF to carbamide-ammonia fertilizer mixtures. The yield increase from their use in the 100 kg/ha ratio (1 : 1) was 0.67 t/ha. The use rate of LCF reduced by half (in the ratio of 1 : 0.5) did not affect the yield of winter wheat. This result demonstrates the effectiveness of complex liquid fertilizers, even at a reduced application rate [9, 11–13].

Pyrophosphoric acid salts do not interact chemically with soil calcium and magnesium carbonate. Therefore, the redistribution of carbonates in the top layer can be achieved by selecting the method of its treatment, namely, the most common method of surface or no tillage. Many studies in China also confirm this [14–17].

Thus, the data obtained in 2018–2020 [17, 18] showed that the use of LCFs together with no-tillage was effective in improving physicochemical and microbiological soil properties. Furthermore, it directly affected crop quality, nutrient availability, and photosynthesis in citrus, apples, tea, and rice plantations. In addition, there was also a tendency to reduce the pollution of agroproducts and the environment by NO_3_.

The newest study was performed with new fertilizing complexes in the form of organic and inorganic compounds [16]. Namely, a complex containing 10% organic matter, 26% urea, 50% superphosphate, 12% potassium chloride, and 1.9% zinc sulfate was applied in the amount of 900 kg/ha. The monitoring of yield formation and biosynthesis of 2-acetyl-1-pyrroline was also performed in a four-year field experiment with aromatic rice (*Orýza Basmati*). When treated with this fertilizer complex, grain yield ranged from 5.86 to 8.29 t/ha and was significantly higher than when treated with simple fertilizers. Increased yield of crops was due to the influence of micronutrient complex on the formation of effective panicles and the rate of seeding. Scientists have also noticed high chlorophyll content and the net productivity of photosynthesis, as well as an increase in the aboveground biomass of aromatic rice. Compared to simple fertilizers, the protein and amino acids of proline and 1-pyrroline in grain doubled from 21% to 38%.

## 3. Task Statements

In many studies in this direction, considerable attention is given to the effect of macronutrients on the yield and quality components of agrocrops, biochemical indicators of photosynthesis, protein composition, and physical and biological properties [12, 19–25]. However, there are practically no works that comprehensively reveal the impact of macro- and microelements of complex fertilizers on soil's chemical composition, the activity of antioxidant enzymes, and CO_2_ gas (an odorless and colorless gas, precisely the source of pure carbon for plants, which is fundamental to all their life processes) exchange.

This study also emphasizes the negative impact of LCF on soil chemistry and biophysical properties. In the current situation with soil exhaustion, fertilizers harm the mineral balance of the soils, the properties, and the land fertility.

Nitrogen compounds significantly acidify the soil, and a large amount of fluorine and potassium disturb macro-and micronutrient food crops, and their water balance, causing a variety of fungal diseases and damage by pests. Poor quality of complex fertilizer mixtures (unbalanced composition and excessive doses) leads to nitrate pollution of products and soils.

The hypothesis of the study is that the use of complex fertilizers on heavy loam soils in grey forests can increase their fertility and improve some physical and chemical properties of the soil. In this regard, the study's objective is to understand how different LCFs affect the agroproperties of heavy clay soils in grey forests of Russia compared with the application of individual elements of soil nutrition.

The following tasks were formulated in order to achieve the aim of the research:

1. To define the influence of the mineral fertilizer application on soil acidity.
2. To establish the influence of complex fertilizers on changes in the content of nitrogen and phosphorus compounds accessible for plants.
3. To identify a change in humic acid content in soil samples with complex fertilizers application.
4. To determine the influence of fertilizers' correlation dependence of crop yield on the nitrate nitrogen (the natural form of nitrogen in the soil, created through nitrification, the conversion of ammonia to nitrate) content in the soil.
5. To evaluate the influence of complex fertilizers on soil CO_2_ exchange in the soil.

## 4. Materials and Methods

### 4.1. Experimental Design

The study is based on a four-year field experiment to determine the impact of complex fertilizers on the nutrient regime of grey forests in heavy loam soils, the processes of plant growth and development, and the formation of fodder beet yields.

The model-laboratory method of biotesting was used to evaluate the biological activity, phytotoxicity, and efficiency of the fertilizers under study. This study also used laboratory and analytical methods to determine the agrochemical composition of fertilizers, soil, and plants, as well as statistical analysis to establish the reliability of the experimental data.

The study of a multifactor experiment was a part of the field experiment on an area covered by grey forests in 2018-2021.

**Figure 1:**
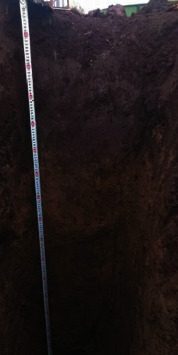
Profile structure of grey forest loamy forest soil (arable land).

The soil profile structure of the grey forest loam (arable land) can be seen in Figure 1.

The general characteristics and agrochemical composition of the soil before the experiment are given in Table 1.

### 4.2. Soil Preparation and Fertilization

The plot for 2018–2021 crops alternation, fertilizer system, and agrotreatment of soil is the same. Unified agrotechnical methods were applied to the plot and reconnaissance sowing was carried out.

Sown winter triticale (Triticosecale Witt.), variety one (“Hermes”), agrotechniques of the establishment of experience, and treatment at all experimental plots were the same. Treatment against pests, diseases, and weeds throughout the experimental field was done with the same preparations (one dose, one technique).

Studies were carried out in a seven-field grain and fallow crop rotation with the following alternation of crops:

1. Winter rye (s/m) + annual herbs
2. Winter triticale
3. Perennial herbs
4. Winter rye
5. Fodder beets
6. Flax
7. Corn + lupine for grain

There were 55 plants before harvesting from the yield structuring variant. Cold-resistant fodder beet *(Beta vulgaris L*. subsp. vulgaris var. crassa) of adaptive variety (Eckendorf yellow) was an experimental crop. It was a medium-ripening variety with a vegetation period of 140–150 days. The average yield was 148 cwt/ha.

The schemes of experiments included mineral fertilizers NH_4_NO_3_ (Naa 34.5%), (NH_2_)_2_CO (Nm 45%), complex fertilizer KAS-32 (35% carbamide, 45% ammonium nitrate, 20% water) with potassium sulfate (K2SO4 46%), diammophoska (DAFK, 10/25/25), “Akvaren 3” (3/11/35), and “Superagro” N/P/S (10/40/5).

The soil treatment did not include tillage. Before sowing, the experimental plot was cultivated with a heavy disc harrow, and seed sowing was carried out by an STVS-18 seeder.

The model experiment in the form of a vegetation experiment was carried out according to the standard technique in metal vessels with a soil weight of soil 300 g (drainage and gas drainage tube are standard in the vessel). The vegetation experiment was laid in PVC vessels with a 10 kg/vessel soil mass and a vessel size 30 × 30 cm.

All experiments were conducted in 4-fold replication. Fertilizers were applied as solutions of 10 ml per 0.1 g of nutrient (pipettes with burettes were used for solutions over 50 ml measuring cylinders).

Experiment diagrams are as follows:

1. RK (background—biofertilizers—control). The biofertilizer utilized in this study is the Natural Microbial Complex Universal (NMC-U). This bacterial concentrated liquid fertilizer is designed to enrich the agroecosystem of soils with beneficial microbiota. The dominance (prevalence) of beneficial microorganisms in soil microflora contributes to the enhancement of plant root nutrition, stimulation of plant growth, and development and increases their resistance to diseases and stress factors of various origins
2. RK + NH_4_NO_3_
3. RK + (NH_2_)_2_CO
4. RK + KАS-32
5. RK + DAFK
6. РK + «Akvaren 3»
7. РK + «Superagro»

In the samples, the soil was evaluated according to a number of indicators as per standards:

- Total nitrogen by Kjeldahl (State Standard (GOST 26107-84))
- Ammonium nitrogen—by Nessler with reagent (K_2_(HgI_4_))
- Nitrate nitrogen—ionometrically.
- Mobile forms of phosphorus and potassium in soil—by the Kirsanov method in modification of CINAO (GOST R 54650-2011)
- Hydrolytic acidity—according to Kappen's modification of CINAO (GOST 26212-91)
- Prepared salt extract (1.0 N KCl) and pH determination—by the CINAO method (GOST 26483-85)
- A sum of absorbed bases—by Kappen–Hilkovitz (GOST 27821-88)
- Determination of humus in soil—by the method of I.V. Tyurin CINAO (GOST 26213-84)
- Determination of fulvic acid by the oxidimetric method (GOST 27980-88 p. 3)
- Humic acids were determined with an extraction method, followed by a spectrophotometric analysis of the obtained extract. This method depends on the ability of humic acids to absorb light in the ultraviolet region of the spectrum. This feature makes it possible to measure their content according to a certain formula using the EC-stink coefficient
- Soil moisture is determined by the gravimetry method: the soil is dried in a dryer at a temperature of 105–110°C to a constant weight. The difference between the initial and final weight of the soil determines the water content of the soil as a percentage
- Soil respiration intensity in standardized environmental conditions identified by the Makarov method
- Soil respiration intensity in standardized environmental conditions determined by the Makarov method

Spectrophotometry (the Shimadzu spectrophotometer (Shimadzu Corporation, Japan)) identified the nitrate nitrogen content in root crops.

### 4.3. Statistical Analysis

All experiments were repeated three times for each variant of the intervention. The obtained research results were processed for reliability using the MANOVA multivariate variance analysis using the Microsoft Excel software and the Statistica 10 software package. Differences in the results obtained are possible at a significance level of *P*=0.05 according to Student's criterion.

### 4.4. Study Limitations

The limitation of this study lies in its restricted geographical representativeness, as the research findings may not comprehensively reflect the diversity of soils and climates across various regions of Russia or other countries.

## 5. Results and Discussion

**Figure 2:**
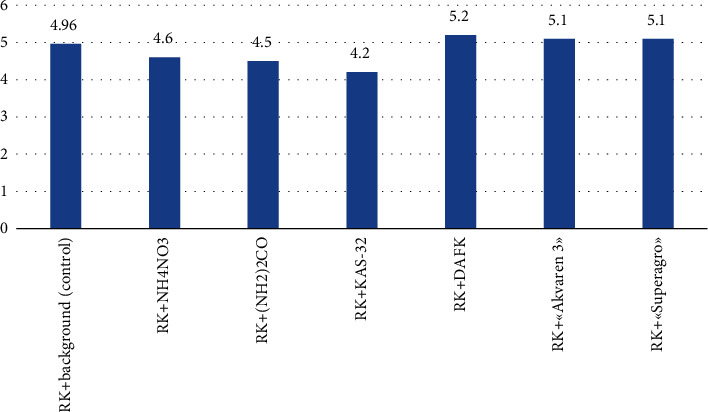
Impact of fertilizers on soil acidity.

Several soil parameters constantly change when applying mineral fertilizers. Those parameters included exchangeable soil acidity and nitrate nitrogen content. In grey forest heavy loam soil, the acidity reaction of the medium is acidic pH = 4.5–5 [26, 27]. Experiments with the application of complex fertilizers DAFK, “Akvaren 3”, and “Superagro” showed a slight decrease in soil acidity (up to 2%). Complex fertilizers KAS-32 with the addition of K_2_SO_4_ facilitate the soil acidity even more (up to 4—5%), indicating the study's results (Figure 2).

Therefore, based on the obtained data, it can be inferred that the application of fertilizers induces changes in soil acidity. The introduction of fertilizers RK + NH_4_NO_3_, RK + (NH_2_)_2_CO, RK + KAS-32, and RK + “DAFK” leads to a decrease in soil pH towards more acidic conditions, whereas fertilizers RK + “Akvaren 3” and RK + “Superagro” exert minimal influence on soil acidity.

Grey forest soils are relatively rich in gross phosphorus, but most of it is in a passive state for the roots of plants. Field experiments had shown that before the experiments, the soils received phosphorus or organic fertilizers, so in the arable layer, acid-soluble P_2_O_5_ (by Kirsanov) is at 59 mg/kg, while according to the reference data for uncultivated soils indicator makes 5–10 mg/kg. However, some subtypes of grey forest soils with a high content of acid-soluble phosphorus are not caused by soil fertility. Therefore, it is possible to use phosphorus fertilizers on these soils.

It was proven that local soils are provided with mobile phosphates since studies of fodder beets found a significant removal of phosphorus in the root crop (up to 26% of the total removal with the harvest). Nevertheless, the content of phosphorus in the soil has not changed. It is caused by using complex fertilizers, such as “Akvaren 3”, phosphate, that can be found in the soil even after harvest. However, the amount of exchange potassium in the soil has risen after using this complex fertilizer (an average of 8%). There is a high content of easily hydrolyzable nitrogen in the arable layer of grey forest soils, about 95 mg/kg. This feature makes heavy loam soils in grey forests similar to chernozems, providing high fertility of the land.

Table 2 presents the characteristics of the studied soil.

Vegetation experience with systematic irrigation and maintaining optimum temperature created conditions for productive vegetation and significant nutrient saturation of root crops with nitrate nitrogen. In the control experiment, the lowest nitrogen content was observed in the crop soil (11.5 mg/kg). With the application of “Akvaren 3” and KAS-32 with different nitrogen content, the accumulation of nitrate nitrogen in the soil did not differ significantly. Nevertheless, the highest observed root crop yield amounted to 150.1 t/ha. Despite the high nitrogen content in the biomass of fodder beet, the mineral nitrogen content in the soil by the end of the growing season was significant. The experiment with urea (NH_2_)_2_CO (Nm 45%) received the most significant volume of nitrogen (gross and nitrate), as this fertilizer contains the highest % of N among all samples. That led to the accumulation of nitrate nitrogen in root crops (up to 12% of the total yield removal).

Experiments with the complex fertilizers diammophoska and “Akvaren 3” show a high level of transition of these chemical elements in the soil (up to 22%) after the end of the growing season. It can be explained by the high content of potassium and phosphorus in these fertilizers and the intense destruction of the crystal lattice of potassium and phosphorus gross forms under the influence of temperature and moisture conditions.

Analysis of soil for ammonium nitrogen showed a reduced result in all experiments except for the experiment with ammonium nitrate (NH_4_NO_3_). The experiment with ammonium nitrate with background (biofertilizer) showed a reliable result of 2.5 mg/kg. It proves that the use of complex fertilizers significantly affects the intensity of soil mineralization compared to individual simple fertilizers, especially in assimilating various forms of nitrogen. The crop's biomass assimilates almost all the nitrogen, but the nitrogen depletion of the soil does not increase significantly (fluctuations of indicators at 3-4%).

Overall, the application of different fertilizers influenced the nitrogen, phosphorus, and potassium content in the soil. Fertilizers RK + KAS-32 and RK + “Superagro” demonstrated the greatest positive impact on the content of all three elements, while fertilizer RK + Fon exhibited the lowest values. These findings underscore the importance of selecting appropriate fertilizers to improve nutrient content in the soil.

In the study of the humic acid content in the soil samples with the complex fertilizers with high nitrogen content KAS-32, ammonium nitrate, and urea, the increase of the mobility of some humic acids in the upper layers compared with the control variant with the background of biofertilizers is noted, for example, the content of fulvic acids increased to 15%. Thus, 1.11 mg/kg of fulvic acid was detected in the control soil. In the variant with urea at 1.45 mg/kg, when adding KAS-32, it increased to 1.75 mg/kg. It indicates that complex fertilizers can improve the quality of humus, replenish its reserves, and reduce nitrate nitrogen content in grey forest soils with heavy loam. The fertilizers contribute to the decomposition of mobile and stable forms of nitrogen humus compounds.

This study shows the dependence between the yield of fodder beet and the nitrate nitrogen content in the soil (Table 3).

The results demonstrate changes in the content of humic acids in soil samples following the application of complex fertilizers. Fertilizer RK + KAS-32 exhibits the highest content of humic acids, reaching 25.4%. This finding suggests the potential effectiveness of this fertilizer in stimulating the formation and accumulation of humus in the soil.

Fertilizers RK + DAFK and RK + “Akvaren 3” also exhibit relatively high content of humic acids—22.4% and 23.2%, respectively. This indicates their potential influence on humus formation and the improvement of soil fertility. Fertilizers RK + NH_4_NO_3_ and RK + (NH_2_)_2_CO also show elevated levels of humic acids—15.6% and 18.2%, respectively.

Therefore, the application of complex fertilizers with different compositions contributes to an increase in the content of humic acids in the soil, which can positively impact soil fertility and quality.

This study shows the dependence between the yield of fodder beet and the nitrate nitrogen content in the soil (Table 4).

Correlation coefficient:

(1) $$\begin{array}{c} r=\frac{\sum \left(X-\bar{x}\right)\left(Y-\bar{y}\right)}{\sqrt{\sum {\left(X-\bar{x}\right)}^{2}\sum {\left(Y-\bar{y}\right)}^{2}}}\\ =\frac{1,030.44}{\sqrt{364.19\cdot 3,948.9}}\\ =0.90. \end{array}$$

At *r* = 0.8, there is a significant correlation relationship.

The standard error for estimating the reliability of the correlation coefficient:

(2) $$\begin{array}{c} S_{r}=\sqrt{\frac{\left(1-r^{2}\right)}{\left(n-2\right)}}\\ =\sqrt{\frac{\left(1-0.86^{2}\right)}{\left(4-2\right)}}\\ =0.4. \end{array}$$

Determination coefficient:

(3) $$\begin{array}{c} d_{\text{yx}},\%=r\times 100\\ =0.90\times 100\\ =90\%. \end{array}$$

Linear regression equation:

(4) $$\begin{array}{c} Y\left(1\right)=y+b_{\text{yx}}\left(X-\bar{x}\right)\\ =90.1+2.8\left(-10.25\right)\\ =61.4, \end{array}$$

where *b*_yx_–regression coefficient:

(5) $$\begin{array}{c} b_{\text{yx}}=\frac{\sum \left(X-\bar{x}\right)\left(Y-\bar{y}\right)}{\sum {\left(X-\bar{x}\right)}^{2}}\\ =\frac{1030,44}{364,19}\\ =2,8. \end{array}$$

**Figure 3:**
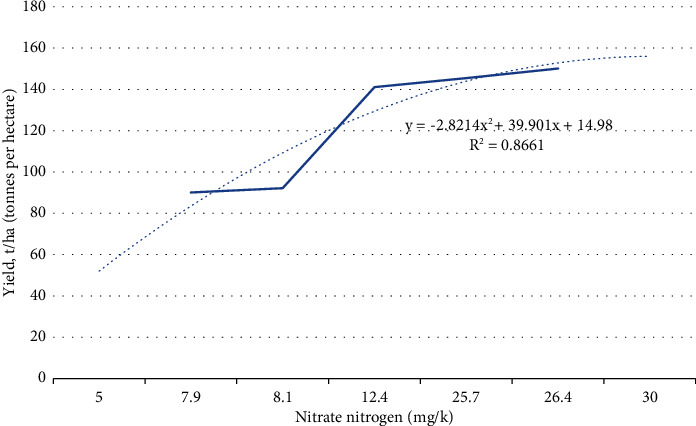
Correlation dependence of fodder beet yield on nitrate nitrogen content in the soil, *r* = 0.8661.

Figure 3 shows the graph of the correlation dependence of fodder beet yield on nitrate nitrogen content in the soil.

A decrease in the mobility of humic acids and the depletion of nitrate nitrogen reserves can harm soil fertility and crop yields. Experiments revealed a direct correlation (*r* = 0.8661) between the nitrate nitrogen content in the soil and the yield of fodder beet, making it possible to predict the yield of root crops.

The global processes of organic matter decomposition in soils and the vital activity of microorganisms cause the annual release of 220 billion tons of carbon dioxide into the atmosphere [21].

The rate of production and release of CO_2_ by the soil is an important indicator that determines the intensity of soil organic matter mineralization when applying fertilizers. All samples were tested for soil respiration, i.e., CO_2_ generation. The studies were conducted on the third day after fertilizer application and the experiment was conducted in fourfold repetition. Soil samples were examined on saprotrophic soil microorganisms' (microorganisms that feed on dead organic residues, and participate in the mineralization of organic substances—ammonification, nitrification, and nitrogen fixation) carbon dioxide emission intensity.

The amount of CO_2_ emitted by the soil is a consequence of the active work of microorganisms and the biological factor of root respiration as the main agricultural crop and other plants. Weather conditions also have a significant impact on the studied indicator, so the indicators of CO_2_ release from the soil surface during the day fluctuated, and the intensity of CO_2_ release was about 14%. Considering that soil humidity significantly influences CO_2_ production, the moisture content was kept within 18–20% of the total soil mass in all samples in the experiments.

The experiments determined roots' contribution to the total volume of CO_2_ emitted. During the year, this indicator varied from 2% to 52% depending on the time of the year, type of cenosis, and soil moisture. Thus, the CO_2_ emission was up to 40% during the warm period of the year and 27% during the cold period.

**Figure 4:**
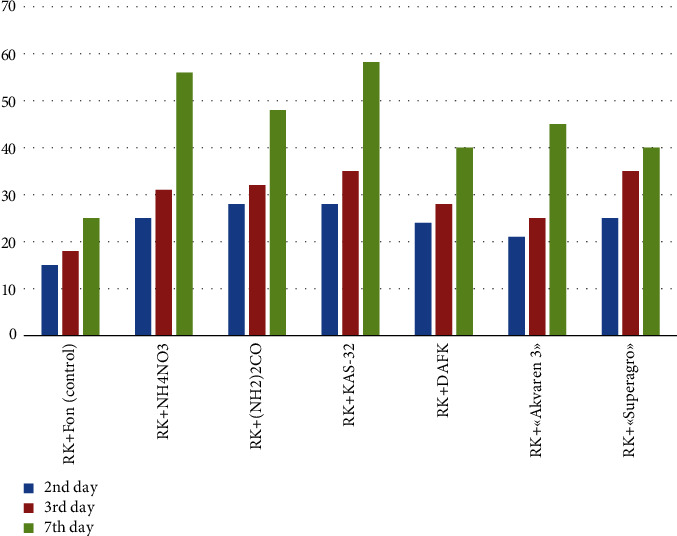
Levels of CO_2_ emissions on the 2nd, 3rd, and 7th days (%).

Figure 4 illustrates the CO_2_ emissions of all samples on the study's 2nd, 3rd, and 7th days. The dynamics were compared with previously obtained data in the scientific literature [12, 26].

In all soil samples, there was an increase in CO_2_ formation on the 2-3 days of the experiment. In the samples with high nitrogen content, ammonium nitrate NH_4_NO_3_ (Naa 34.5%), urea (NH_2_)_2_CO (Nm 45%), and KAS-32 (35% carbamide, 45% ammonium nitrate, and 20% water) showed the most intensive soil respiration as compared with biofertilizer application in the control sample.

The peak values of CO_2_ allocation were noted on the 7th incubation day. The increase of soil respiration was 58.2% in the sample of KAS-32 with potassium sulfate. Since the studies took place in quadruplicate, they are statistically valid.

Measurements showed that the intensity of soil respiration of the control sample (without fertilizers) was, on average, 13 gCO_2_/m^2^/day. After introducing high doses of nitrogen, “Akvaren 3” soil respiration was markedly lower (6.8 gCO_2_/m^2^/day), and it was 11 gCO_2_/m^2^ per day in the experiment with NH_4_NO_3_. The experiment with KAS-32 resulted in 6.5 gCO_2_/m^2^ per day. A decrease in respiration is an adaptation of soil to an imbalance of the C : N ratio. In nature, the C : N ratio is regulated by changing the biological activity of saprotrophic soil microorganisms.

Thus, the study shows that the lowest ability and activity to release CO_2_ from the soil occurs when using biofertilizers, compared with complex fertilizers. They contribute significantly to the emission of carbon dioxide into the atmosphere, negatively affecting the environment.

It was found that the liquid fertilizers KAS-32 and Akvaren-3 do not have a negative effect on the biological activity of the soil. In other variants of the experiments, individual differences in the level of respiratory activity depended on the type of fertilizer. Presumably, it is connected with different contents of the main nitrogen forms (nitrate, amide, and ammonia). The influence of these forms on the metabolism of prototrophic soil microorganisms also varied.

A group of scientists from Ecuador compared the effects of biofertilizers, complex fertilizers, and cultivation methods on soil respiration in chernozem soil. The scientists noted that the indicator of soil microbial activity is CO_2_ emission [28]. This research aims to optimize carbon dioxide emissions in agriculture and improve carbon sequestration. Comparing simple biofertilizer and complex fertilizer in four doses (N60P45K45; N120P90K90; N180P135K135; and N240P180K180) demonstrated reduced soil respiration when applying biofertilizers compared to complex fertilizer. Thus, biofertilizers reduce CO_2_ emissions into the environment. This conclusion was also confirmed by our study on grey forest heavy loam soil. It indicates a similar effect of complex mineral fertilizers on the respiration of different types of soils.

Study data on fertilizers to improve soil health from the International Fertilizer Association in France indicate the change in average crop yields and soil organic carbon content after applying complex mineral fertilizers in a 9-year experiment on irrigated fields in northern India. The organic carbon content in the soil's arable layer (0–15 cm) increased from 14.10 t/ha to 18.62 t/ha after 9 years of using complex fertilizers at a dose of N_120_P_26_K_33_ [29]. This study indirectly confirmed improved soil quality after using complex fertilizers due to the increased availability of humus acids and active forms of nitrogen, phosphorus, and potassium to plants. However, the identified pollution of crops by nitrates when using complex fertilizers confirms the thesis of this study on the need for controlled use of mineral fertilizers. In this case, it is also crucial to consider both the economic and practical benefits and the detrimental effects on ecosystems.

Studies by many scientists [19, 30–33] showed a similar relationship between the chemical and physical properties of soils and quantitative yield assessment under the influence of complex fertilizers. Experiments by Maltas et al. [34] (Switzerland) and Hirzel et al. [21] conducted under controlled conditions in a Chile laboratory indicate that the application of organic fertilizers had different effects on soil pH, soil salinity, and concentrations of phosphorus, potassium, calcium, magnesium, and sulfur in loamy and sandy loam soils. When using complex fertilizers, the level of CO_2_ release and the increase in soil acidity were the highest compared to simple biofertilizers. The availability and exchange of K, Ca, and Mg were also noted in experiments with NPK fertilizers. In addition, studies of both soil types have shown differences in assessing changes in chemical parameters over time. The authors attribute these changes to certain initial properties of each soil. The present study also confirmed that the original content of active substances in soils (bottom) qualitatively affects the results obtained for complex mineral fertilizers. These findings have a practical value for calculating doses of mineral components.

According to Alzamel et al. [35], complex fertilizers can induce changes in soil pH, which can have positive and negative effects. For instance, some complex fertilizers may have an alkaline nature, leading to an increase in pH, which is beneficial for reducing acidity in acidic soils. However, other fertilizers may be acidic and contribute to a decrease in soil pH. Therefore, the selection of fertilizers should consider the initial soil acidity level and the objectives of improving its properties [35].

Research conducted by Gao et al. [36] demonstrate that the utilization of complex fertilizers can contribute to more efficient utilization and absorption of phosphorus, potassium, and nitrogen by plants. However, factors such as soil type, climatic conditions, fertilizer dosage, and application methods need to be considered to achieve optimal results and avoid potential negative consequences associated with excessive fertilizer application [36].

Bilong et al. [37] also note that a balanced combination of urea and compost can reduce NH_3_ volatilization and enhance soil fertility. According to the author's research, the application of compost (5000 kg·ha^−1^) with urea at a rate of 100 kg·N ha^−1^ or 200 kg·N·ha^−1^ is recommended to reduce NH3 volatilization, inhibit nitrification, and effectively retain NH4+ in the soil, thereby influencing soil fertility [37].

The results of the scientific work entitled “The Effect of Complex Fertilizers Used for Early Maturing Potato Crop on Sandy Loam Soil” by Wadas and Dziugieł [38] confirm this study's data on the root crop and fodder beet. The productive effects of complex fertilizers in early maturing potato crops on loam sandy soils in eastern Poland were comparable to those of single-element fertilizers. The greatest increase in total and marketable tuber yield compared to no-fertilizer crop was observed using nitrofoska with the lowest concentration of N-NH_4_. Root crops and potatoes are highly sensitive to NH_4_ and changes in soil pH. When applied repeatedly, fertilizers with high ammonium nitrogen content acidify the soil. Soil microorganisms transform ammonium nitrogen into NO_3_, releasing H ions that acidify the soil. The high agronomic efficacy of introducing complex nitrogen fertilizers, such as KAS-32, is demonstrated in cultivating very early maturing potatoes on loam and sandy soils. These fertilizers provide good nutrition and improve the water supply for root crops.

Research indicates that the application of complex fertilizers can contribute to an increase in the content of humic acids in the soil. Humic acids positively influence the soil's aggregate structure, promoting the formation and stabilization of soil aggregates. This enhances soil water-holding capacity, permeability, and air exchange. Humic acids serve as natural chelating agents, facilitating the uptake and mobilization of micronutrients in the soil. Complex fertilizers containing humic acids can enhance the availability of nutrients for plants, thereby improving soil fertility and plant growth, as also observed in the studies conducted [39, 40].

In trials assessing nutrient release, Singh and Ryan [29] demonstrated that the use of complex fertilizers allows for the control and deceleration of nutrient release. This approach minimizes nutrient losses due to leaching into lower soil layers and groundwater, thus preventing groundwater contamination, particularly by nitrates [29].

Thus, the novelty of the study is that it covers the effect of complex fertilizers on a specific type of soil. Consequently, the findings may be useful for improving agricultural production and soil conservation. In addition, the study of the effect of complex fertilizers on the properties of heavy loam soils in grey forests is an important direction. The research on this issue may produce useful practical recommendations for improving soil quality and increasing yields.

## 6. Conclusions

Аnalyzing nitrogen (N), phosphorus (P_2_O_5_), and potassium (K_2_O) content in the soil following the application of various fertilizers, the following conclusions can be drawn:

(1) Nitrogen (N) content in the soil:
  - Fertilizers RK + NH_4_NO_3_, RK+(NH_2_)_2_CO, RK + DAFK, RK + “Akvaren 3”, and RK + “Superagro” led to an increase in nitrogen content compared to the control (RK + Fon).
  - Fertilizers RK + KAS-32 and RK + Fon exhibited lower nitrogen content compared to other fertilizers.
(2) Phosphorus (P_2_O_5_) content in the soil:
  - Application of fertilizers RK + KAS-32, RK + DAFK, RK + “Akvaren 3”, and RK + “Superagro” resulted in an increase in phosphorus content in the soil compared to the control.
  - Fertilizers RK + control, RK + NH_4_NO_3_, and RK + (NH_2_)_2_CO exhibited lower phosphorus content compared to other fertilizers.
(3) Potassium (K_2_O) content in the soil:
  - Fertilizers RK + “Superagro” and RK + KAS-32 resulted in an increase in potassium content in the soil compared to the control and other fertilizers.
  - Fertilizers RK + control, RK + NH_4_NO_3_, RK + (NH2)_2_CO, RK + DAFK, and RK + “Akvaren 3” also exhibited some increase in potassium content, albeit to a lesser extent.

Therefore, the conducted study has revealed several important conclusions:

1. Regulation of soil parameters: The use of complex DAFK fertilizers contributes to a moderate reduction in soil acidity, which may be beneficial for creating optimal conditions for plant growth.
2. Nitrogen efficiency: The application of urea fertilizers leads to nitrogen accumulation in root crops, providing an opportunity to optimize fertilizer use and manage nitrogen content in the soil.
3. Transition of chemical elements: The intensive transition of chemical elements in the soil occurs after the vegetation period, which can enhance soil fertility.
4. Humus and nitrate nitrogen: Fertilizers such as “Diammophoska” and “Akvaren 3” promote increased mobility of humic acids, improving humus quality and reducing nitrate nitrogen content in the soil.
5. Crop forecasting: A direct correlation has been found between nitrate nitrogen content and fodder beet yield, which can aid in crop forecasting.
6. Influence on soil respiration: The use of different fertilizers affects soil respiration intensity, which may be associated with soil adaptation to carbon-nitrogen imbalance.

The study on the impact of complex fertilizers on soil provides valuable scientific foundations for practical application in agriculture. The results indicate the necessity of regulating fertilizer composition to enhance their effectiveness, potentially leading to increased crop yields and improved soil quality. The development of nitrogen management methods, grounded in the findings of this research, becomes a key tool for optimizing nitrogen use and preventing negative environmental impacts.

Further research in this field may focus on expanding knowledge and deepening the understanding of fertilizer effects on soil processes. Specifically, investigating the interaction of fertilizers with soil microorganisms and their role in biological activity and soil fertility could be a focal point for future studies.

## Figures and Tables

**Table 1:** Agrochemical characteristics of heavy loam soils in grey forests.

Horizon (cm)	Humus (%)	рН_(KCl)_	Нg	S	ЕKО	Degree of base saturation V (%)	Р_2_O_5_	K_2_О
			mg equivalent/100 g soil				mg/kg of soil	
A_max_ (0–12)	8.14	4.96	6.26	11.2	40.92	82.8	109	102
A_1_ (12–36)	6.50	4.98	7.16	10.4	35.36	81	88	96
A_1_A_2_ (36–58)	5.19	4.81	4.91	9.8	33.72	83.9	71	72
A_2_В (58–85)	2.70	4.72	3.20	10.8	28.23	81.7	65	68
B_1_ (85–143)	0.41	4.33	2.42	9.4	28.91	83.0	61	51
B_2_ (143–160)	0.19	4.47	2.15	8.8	29.31	—	—	—

**Table 2:** Characteristics of the grey forest heavy loam soils, data for 2021.

Horizon (cm)	Mobile (mg/kg)			Gross (%)		
	Р_2_О_5_	K_2_О	N-NO_3_	N	Р_2_О_5_	K_2_О
	By kirsanov					
RK + Background (control)	109	102	11.5	0.165	0.415	1.074
RK + NH_4_NO_3_	112	100	15.6	0.198	0.408	1.065
RK + (NH_2_)_2_CO	115	112	18.2	0.205	0.398	1.055
RK + KAS-32	124	120	32.4	0.211	0.401	1.060
RK + DAFK	398	241	25.4	0.175	0.454	1.120
RK + “Akvaren 3”	450	300	16.2	0.170	0.422	1.115
RK + “Superagro”	456	285	15.2	0.155	0.501	1.095

**Table 3:** Influence of fertilizers on the content of humic acids (%).

Fertilizer	Content of humic acids (%)
RK + Fon (control)	11.5
RK + NH_4_NO_3_	15.6
RK + (NH_2_)_2_CO	18.2
RK + KAS-32	25.4
RK + DAFK	22.4
RK + “Akvaren 3”	23.2

**Table 4:** Determination of correlation coefficient between yield of fodder beet and the amount of nitrate nitrogen in the soil.

Experiment	Deviation from average		Squares of deviations		Sum
1	−10.25	−42.1	105.0625	1,772.41	431.525
2	−10.05	−40	101.0025	1600	402
3	−5.75	8.9	33.0625	79.21	−51.175
4	7.55	13.3	57.0025	176.89	100.415
5	8.25	17.9	68.0625	320.41	147.675
Average	—	—	364.1925	3,948.92	1,030.44

## Data Availability

The data used to support the findings of this study are available upon reasonable request.
